# Supplementary material for: Mutations in PCYT2 disrupt etherlipid biosynthesis and cause a complex hereditary spastic paraplegia
Source: Brain. 2019 Oct 22;142(11):3382–97. doi: 10.1093/brain/awz291 (PMC6821184; doi:10.1093/brain/awz291)
Supplement: awz291_Supplementary_Data [file awz291_supplementary_data.zip › awz291-Suppl_data/Supplementary_Data4.zip!/Supplementary information 3 - fibroblast lipidomics.zip!/Fibroblast lipidomics/Patient1 vs Patient2/Statistical plots and data/PA species/Boxplots.pdf]

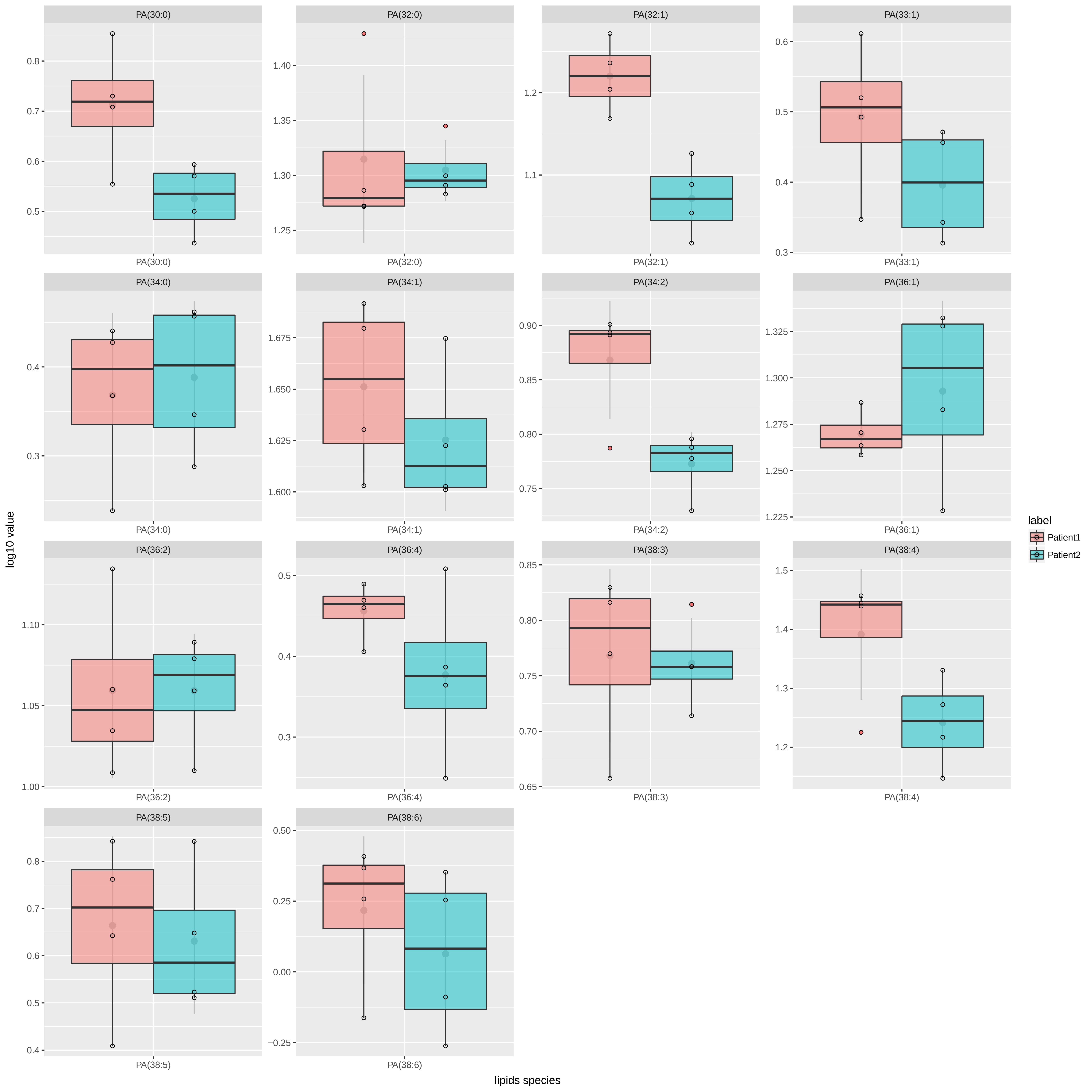
PA(30:0)
PA(32:0)
PA(32:1)
PA(33:1)
PA(34:0)
PA(34:1)
PA(34:2)
PA(36:1)
PA(36:2)
PA(36:4)
PA(38:3)
PA(38:4)
PA(38:5)
PA(38:6)
log10 value
lipids species
label
Patient1
Patient2
